# Expresión de los componentes del inflamasoma y su relación con los marcadores de riesgo cardiovascular en personas con infección por HIV-1

**DOI:** 10.7705/biomedica.6320

**Published:** 2022-06-01

**Authors:** Kevin León, Damariz Marín-Palma, Salomón Gallego, Cristina Yepes, Jhonatan Vélez, Gustavo A. Castro, Fabián Jaimes, Natalia Taborda, María Teresa Rugeles, Juan C. Hernández

**Affiliations:** 1 Infettare, Facultad de Medicina, Universidad Cooperativa de Colombia, Medellín, Colombia Universidad Cooperativa de Colombia Infettare, Facultad de Medicina Universidad Cooperativa de Colombia Medellín Colombia; 2 Grupo de Inmunovirología, Facultad de Medicina, Universidad de Antioquia, Medellín, Colombia Universidad de Antioquia Grupo de Inmunovirología Facultad de Medicina Universidad de Antioquia Medellín Colombia; 3 Grupo de Investigaciones Biomédicas Uniremington, Programa de Medicina, Facultad de Ciencias de la Salud, Corporación Universitaria Remington, Medellín, Colombia Corporación Universitaria Remington Grupo de Investigaciones Biomédicas Uniremington, Programa de Medicina Facultad de Ciencias de la Salud Corporación Universitaria Remington Medellín Colombia; 4 Dirección de Investigaciones, Fundación Hospitalaria San Vicente de Paúl, Medellín, Colombia Dirección de Investigaciones Fundación Hospitalaria San Vicente de Paúl Medellín Colombia; 5 Departamento de Medicina Interna, Facultad de Medicina, Universidad de Antioquia, Medellín, Colombia Universidad de Antioquia Departamento de Medicina Interna Facultad de Medicina Universidad de Antioquia Medellín Colombia

**Keywords:** HIV-1, inflamasomas, cardiopatías, replicación viral, HIV-1, inflammasomes, heart diseases, virus replication

## Abstract

**Introducción.:**

La infección por el HIV-1 induce un estado de inflamación crónico en el que participan los inflamasomas. El incremento de los parámetros inflamatorios es mayor en individuos con replicación viral activa que en aquellos con control de la replicación viral. Este proceso desencadena alteraciones metabólicas relacionadas con cambios en el perfil lipídico, lo cual podría incrementar el riesgo de eventos cardiovasculares, incluso en pacientes con terapia antirretroviral.

**Objetivo.:**

Establecer si existe correlación entre la expresión de los componentes de los inflamasomas y los marcadores de riesgo cardiovascular en individuos con control de la replicación viral y en aquellos con replicación viral activa con terapia antirretroviral o sin ella.

**Materiales y métodos.:**

Se estudiaron 13 individuos con control de la replicación viral y 40 con replicación viral activa (19 sin terapia antirretroviral y 31 con terapia). Se evaluaron los marcadores clásicos de riesgo cardiovascular y se cuantificó mediante RT-PCR la expresión de los componentes de los inflamasomas (NLRP1, NLRP3, NLRC4, AIM2, ASC, IL-1β, IL-18 y caspasa-1), TLR2, TLR4, TGF-β e IL-10.

**Resultados.:**

Se observó que los pacientes con replicación viral activa y con terapia antirretroviral presentaron un incremento en la expresión de TLR2, TLR4 e IL-18, comparados con los controladores del HIV-1. Además, mostraron grandes valores de triglicéridos y lipoproteína de muy baja densidad (*Very Low Density Lipopretein*, VLDL), lo que se correlaciona positivamente con la expresión de los componentes de los inflamasomas NLRP1, NLRP3, NLRC4, AIM2, ASC y caspasa-1.

**Conclusión.:**

El aumento en la expresión de los componentes de los inflamasomas en los individuos con replicación viral activa y con terapia antirretroviral se correlacionó con las concentraciones de triglicéridos y VLDL, lo que sugiere el papel de la activación inmunitaria y la terapia antirretroviral en el riesgo cardiovascular.

## Abstract (en)

**Introduction:**

HIV-1 infection induces a chronic inflammatory state in which inflammasomes participate. The increase in inflammatory parameters is higher in individuals with active viral replication (progressors) than in those with viral control (HIV-1 controllers). This process triggers metabolic alterations related to changes in the lipid profile, which could increase the risk of cardiovascular events, even in patients with antiretroviral therapy.

**Objective:**

To establish whether there was a correlation between the expression of inflammasome components and cardiovascular risk markers in HIV-1 controllers and progressors with or without antiretroviral therapy.

**Materials and methods:**

We studied 13 HIV-1 controllers and 40 progressors (19 without antiretroviral therapy and 31 with therapy) and evaluated in them classic markers of cardiovascular risk. Using RT-PCR we quantified the expression of inflammasome components (NLRP1, NLRP3, NLRC4, AIM2, ASC, IL-1β, IL-18, and caspase-1), TLR2, TLR4, TGF-β, and IL-10.

**Results:**

Progressors with antiretroviral therapy had an increased expression of TLR2, TLR4, and IL-18 compared to HIV-1 controllers. They also showed high levels of triglycerides and VLDL, which positively correlated with the expression of the inflammasome components NLRP1, NLRP3, NLRC4, AIM2, ASC, and caspase-1.

**Conclusion:**

Progressors receiving antiretroviral therapy exhibited an increased expression of the inflammasome components, which correlated with the levels of triglycerides and VLDL. This supports the role of inflammation in cardiovascular risk during HIV-1 infection.

La infección por el HIV-1 es un problema de salud pública global, pues tiene una carga importante de morbimortalidad. Entre las personas con infección por HIV-1, se encuentran diferentes patrones de progresión de la infección; la mayoría, denominados *"progressors"* típicos (sin control de la replicación viral), desarrolla el síndrome de inmunodeficiencia adquirida (sida) al cabo de 5 a 10 años en ausencia de la terapia antirretroviral combinada (C*ombined Antiretroviral Therapy*, cART). Sin embargo, existe un grupo de denominados “controladores” del HIV-1 (con control de la replicación viral) en ausencia de dicha terapia y mantienen cargas virales menores de 2.000 copias/ml ^1^^,^^2^.

La historia natural de la infección por HIV-1 se caracteriza por el establecimiento de un estado inflamatorio crónico, el cual se asocia con una mayor progresión a sida, especialmente en aquellos individuos que no están recibiendo terapia antirretroviral combinada ^3^. Múltiples mecanismos se asocian con el desarrollo de la activación persistente de la respuesta inmunitaria, entre los que se encuentran la replicación viral activa, la translocación microbiana desde el intestino a la circulación sistémica, la alteración de la microbiota intestinal, las coinfecciones crónicas, la pérdida de células que regulan la respuesta inmunitaria, y el daño generalizado de los tejidos linfoides ^4^^,^^5^. En este sentido, la infección por HIV-1 se asocia con un acentuado incremento en la producción de las citocinas proinflamatorias IL-1β, IL-6 e IL-18 ^6^^,^^7^, algunas de las cuales, como la IL-1β y la IL-18, se han correlacionado directamente con una mayor replicación viral *in vitro*^8^^,^^9^, pues pueden promover la actividad de transcripción del HIV-1, como es el caso de la IL-1β ^10^.

La IL-1β y la IL-18 son citocinas proinflamatorias que requieren de un proceso de maduración proteolítico para adquirir su forma biológicamente activa. Dicho proceso se lleva a cabo principalmente en plataformas multiproteicas denominadas inflamasomas ^11^^,^^12^. Estos complejos presentan receptores citosólicos de la familia de los *NOD-like receptors* (NLR), que hacen parte de un grupo de mediadores importantes de la respuesta inmunitaria innata llamados receptores de reconocimiento de patrones (*Pattern Recognition Receptor*, PRR), entre los cuales se encuentran los receptores de tipo *toll* (TLR) 2 y 4, que se activan gracias a la presencia de estructuras moleculares conservadas en los microorganismos (*Pathogen-Associated Molecular Patterns,* PAMP) o moléculas derivadas del daño causado a las células en diversos procesos inflamatorios (*Damage- Associated Molecular Patterns,* DAMP) ^13^.

Los inflamasomas mejor descritos incluyen el NLRP1, el NLRP3, el NLRC4 y el AIM2, los cuales participan en la maduración proteolítica de citocinas de la familia de la IL-1 ^10^. Estos inflamasomas están conformados comúnmente por una molécula sensora, en la mayoría de ellos un NLR, una proteína adaptadora denominada ASC (*Apoptosis-associated Speck- like protein containing a Caspase activation and recruitment domain*) y la caspasa-1, la cual tiene actividad efectora durante la piroptosis cuando está en su forma biológicamente activa ^14^.

En estudios recientes, se ha sugerido que los inflamasomas podrían jugar un papel importante en la patogénesis de la infección por HIV-1, ya que el virus sirve como primera señal para la activación del inflamasoma NLRP3 por medio de la vía de señalización del NF-κB ^15^. De hecho, previamente demostramos que, comparados con individuos que tienen control de la replicación del HIV-1, aquellos sin este control tienen una mayor expresión de IL-1β, IL-18 y caspasa-1 en células mononucleares de sangre periférica (*Peripheral Blood Mononuclear Cells,* PBMC) y en tejido linfoide asociado con la mucosa del tubo digestivo (G*ut-Associated Lymphoid Tissue,* GALT) ^16^. Además, en quienes no hay control de la replicación viral, se observa un incremento en otros componentes del inflamasoma, como la proteína ASC, la cual se correlaciona directamente con la carga viral ^17^.

Aunque la implementación de la terapia antirretroviral combinada ha aumentado la calidad y expectativa de vida de las personas con infección por HIV-1, actualmente se observa un aumento de otras enfermedades que se originan tanto por los efectos crónicos de la infección como por los efectos adversos del tratamiento. De hecho, se ha reportado que los pacientes con algunos esquemas específicos de la terapia antirretroviral combinada presentan una mayor prevalencia de enfermedades no relacionadas con el sida, entre ellas, alteraciones metabólicas como la dislipidemia, y resistencia a la insulina, lo que a su vez incrementa el riesgo de desarrollar enfermedades cardiovasculares y se relaciona con los efectos descritos de la terapia antirretroviral combinada en el sistema cardiovascular, así como con la activación inmunológica persistente ante la presencia de péptidos virales ^18^^,^^19^.

En el contexto de estas complicaciones, es necesario tener en cuenta las vías metabólicas e inflamatorias implicadas en el aumento del riesgo cardiovascular durante la infección por HIV-1. Diversos biomarcadores tradicionales de riesgo cardiovascular se han asociado con la activación de los inflamasomas ^20^^,^^21^, entre ellos, el colesterol total, las lipoproteínas de alta, baja y muy baja densidad [*High Density Lipoproteins* (HDL), *Low Densitiy Lipoproteins,* (LDL) y *Very Low Densitiy Lipoproteins* (VLDL)] y los triglicéridos. El inflamasoma NLRP3 y algunos otros se han asociado con la formación de placas ateromatosas por aumento en la producción de especies reactivas de oxígeno, disfunción mitocondrial, ruptura de los lisosomas y generación de estrés en el retículo endoplásmico ^22^^,^^23^. Además de las alteraciones en los inflamasomas, las personas con infección por HIV-1 presentan alteraciones en otros marcadores de inflamación relacionados con el desarrollo de las enfermedades cardiovasculares, tales como la proteína C reactiva ultrasensible, la IL-6, el TNF-α, el CD14 soluble, el CD163 soluble y marcadores metabólicos como los niveles de glucosa en sangre, de dímero D y de creatinina ^24^^,^^25^. Sin embargo, aún no es claro el papel que cumplen estos componentes inflamatorios, especialmente los inflamasomas, en el aumento del riesgo cardiovascular en las personas con infección por HIV-1 ^26^^,^^27^.

En el presente estudio, se evaluaron marcadores metabólicos y de riesgo cardiovascular en pacientes con control de la replicación del HIV-1 y sin control, y se evaluó su potencial correlación con la expresión de componentes de los inflamasomas NLRP1, NLRP3, NLRC4 y AIM2.

## Materiales y métodos

### Diseño y población de estudio

Se hizo un estudio transversal analítico de 63 personas con infección por HIV-1-sida en Medellín (Colombia) clasificadas como:

i. individuos con control de la replicación viral (C, n=13) con diagnóstico de infección por HIV-1, por lo menos, un año antes, que no recibían la terapia antirretroviral y tenían una carga viral menor de 2.000 copias/ml ^1^;

ii. individuos sin control de la replicación viral (P, n=19) con evidencia de progresión (recuento de linfocitos T CD4+ menor de 500 células/µl y una carga viral superior a 2.000 copias/ml) ^28^ y que no habían recibido terapia antirretroviral combinada, y

iii. individuos sin control de la replicación viral con terapia (n=31) que en el momento de ingreso al estudio se encontraban bajo terapia antirretroviral combinada, con un recuento de linfocitos T CD4+ mayor de 250 células/µl y carga viral indetectable (<40 copias/ml).

No se incluyeron personas con diagnóstico o historia previa de eventos cardiovasculares o diabetes, ni aquellos con tratamiento inmunomodulador, mujeres gestantes o en lactancia, ni individuos con procesos neoplásicos activos diagnosticados o alguna otra comorbilidad específica que pudiese ocasionar un sesgo en los resultados.

### Toma de las muestras

Se extrajeron 10 ml de sangre periférica por venopunción en tubos Vacutainer con EDTA; a partir de esta, se obtuvieron el plasma y las células mononucleares de sangre periférica por medio de un gradiente de densidad, utilizando Ficoll Histopaque -1077 (Sigma-Aldrich, St. Louis, USA). Estas células se preservaron a -80 °C hasta su procesamiento en TRIzol (Invitrogen, Life Technologies, CA) para su posterior purificación y la extracción de ARN.

### Cuantificación de moléculas inflamatorias mediante PCR en tiempo real

Para la extracción del ARN total se usó Direct-zol RNA MiniPrep™, siguiendo las instrucciones del fabricante. La concentración del ARN se cuantificó por espectrofotometría en un equipo NanoDrop 1000™ (Thermo Scientific, Wilmington, USA).

Se trataron 250 ng de ARN de una pureza entre 1,8 y 2 con DNAsa I, RNase-free™ (Qiagen, Hilden, Germany). La síntesis del ADNc se hizo con el High Capacity cDNA Reverse Transcription Kit™ (Applied Biosystems, Foster City, California, USA), siguiendo las instrucciones del fabricante.

Por último, se cuantificó la expresión génica de los TLR2 y TLR4, de los componentes de los inflamasomas, NLRP3, NLRP1, NLRC4, AIM2, IL-1β, IL- 18, ASC y caspasa-1, y de las citocinas reguladoras IL-10 y TGF-β, mediante PCR en tiempo real y empleando el estuche Maxima SYBR Green qPCR Master Mix (Fermentas, Hanover, MD, USA). Se utilizó el gen constitutivo β**-**actina para la normalización y reporte de unidades relativas de transcriptos (*Relative Transcript Units*, RTU) (cuadro suplementario 1).

### Evaluación de parámetros bioquímicos

Mediante ensayos colorimétricos (Biosystems; Costa Brava 30, Barcelona, España), se evaluaron los siguientes parámetros bioquímicos relacionados con el perfil lipídico: colesterol total, HDL, LDL, VLDL y triglicéridos. Además, se cuantificaron la glucemia en estado basal, el dímero D, la proteína C reactiva ultrasensible y la creatinina, como parámetros adicionales. Estas mediciones se hicieron en un laboratorio clínico certificado de Medellín. Se determinaron los niveles séricos de los siguientes marcadores inmunológicos mediante pruebas inmunoenzimáticas: sCD14 (Human Soluble Cluster of Differentiation 14 (CD14)- MBS2514176_COA) (MyBioSource, San Diego, USA); sCD163 (Human sCD163 Ready-SET-Go 88-50360-22) (Affymetrix eBioscience, Viena, Austria); IL-18 (Human IL-18 Matched Antibody Pairs BMS267/2MST) (eBioscience, Vienna, Austria), e IL-6 (BD OptEIA) (BD Biosciences).

### Análisis estadístico

Debido al escaso número de individuos sin terapia antirretroviral combinada con control o sin control de la replicación viral, no fue posible alcanzar el tamaño de muestra deseado, por lo que la población de estudio se estableció a conveniencia. El análisis estadístico se llevó a cabo usando el paquete estadístico GraphPad Prism 7.0™ (San Diego, CA, USA). Para evaluar la distribución de probabilidades de las variables analizadas (normalidad), se utilizó la prueba de Shapiro-Wilk. Las medias se compararon mediante un análisis de varianza (ANOVA) y, las medianas, con la prueba de Kruskall-Wallis, según correspondiera. Para las correlaciones se emplearon pruebas de Pearson o Spearman. En todos los análisis se consideró un nivel de significación estadística de p<0,05.

### Aspectos éticos

Los participantes del estudio dieron su consentimiento informado y el protocolo fue aprobado por el Comité de Ética de la Universidad Cooperativa de Colombia, sede Medellín (acta: 0800-015). Los aspectos éticos se ajustaron a las normas nacionales e internacionales sobre la confidencialidad de los datos obtenidos y la garantía de la seguridad del paciente, siguiendo los principios éticos de la Declaración de Helsinki, el reporte Belmont y las pautas del CIOMS.

## Resultados

### Características clínicas y sociodemográficas

Se incluyeron 63 personas con infección por HIV-1; el 40,3 % de ellas correspondía a mujeres y, el 59,7 %, a hombres. Los individuos incluidos en el grupo P (sin control de la replicación viral) presentaban recuentos menores de linfocitos T CD4+ y mayores cargas virales, en comparación con aquellos incluidos en los grupos C (con control de la replicación viral) y PT (sin control de la replicación viral y con terapia). El tiempo transcurrido desde el diagnóstico de HIV-1 fue significativamente mayor en el grupo PT que en el P (p<0,001), pero similar al del grupo C. No se observaron diferencias significativas al comparar los grupos C y P. Las principales características de los participantes del estudio se resumen en el cuadro 1.

**Cuadro 1:** Características clínicas y demográficas de la población de estudio

	C (n=13)	P (n=19)	PT (n=31)
Hombres - Mujeres	8 - 6	7 - 7	15 - 10
Edad en años, mediana (Q1-Q3)	29 (25,5 - 45)	30 (25,75 - 34,75)	36 (27,5 - 42,5)
Tiempo de diagnóstico, meses (Q1-Q3)*	42 (31 - 87)	25 (7 - 36)	96 (45 - 139)
LT CD4+ células/µl (Q1-Q3)	859 (523 - 1050)	421 (276 - 538)	648 (363 - 886)
LT CD8+ células/µl (Q1-Q3)	734 (420 - 1808)	823 (307 - 1109)	673 (464 - 970)
Relación CD4/CD8 (Q1-Q3)	1,3 (0,6 - 1,7)	0,5 (0,3 - 1,3)	1(0,8 - 1,2)
Carga viral copias/ml (Q1-Q3)	310 (153 - 731)	22.200 (10.733 - 48.735)	40 (40 - 40)

* P Vs. PT: p<0,001

Los niveles séricos de los marcadores metabólicos (glucemia y creatinina) y de reacción inflamatoria (hs-PCR, dímero-D, sCD14, sCD163, IL-16 e IL- 18) no mostraron diferencias significativas en los grupos evaluados (figura suplementaria 1).

### Expresión de los componentes de los inflamasomas

**Figura 1:**
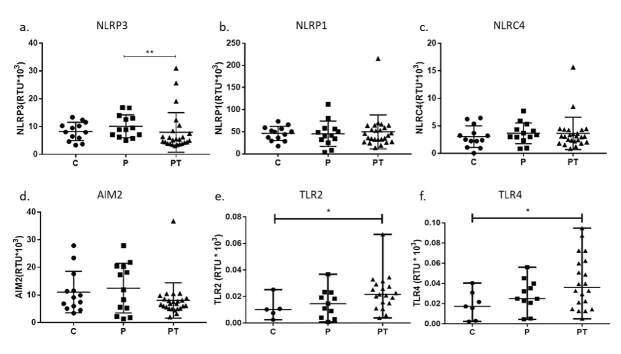
Expresión génica de los componentes de TLR2, TLR4 y de los inflamasomas NLRP1, NLRP3, NLRC4 y AIM2

La expresión de NLRP3 fue significativamente mayor en el grupo PT que en el grupo P (p<0,01). Sin embargo, al comparar los tres grupos de estudio en cuanto a la expresión de NLRP1, NLRC4 y AIM2, no se hallaron diferencias estadísticamente significativas (figura 1A-D). Los individuos del PT presentaron una mayor expresión de TLR2 y TLR4, comparados con los del grupo C (p<0,05) (figura 1E-F).

**Figura 2:**
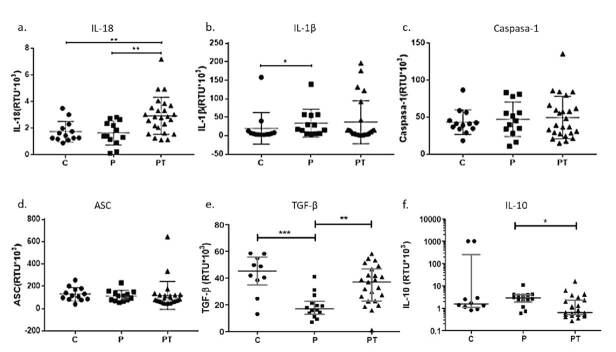
Expresión génica de citocinas reguladoras y proinflamatorias

La expresión de IL-18 fue significativamente elevada en individuos del grupo PT en comparación con los grupos C y P (p<0,01), en tanto que la expresión de IL-1β fue mayor en el grupo P que en el C (p>0,05). Por su parte, la proteína adaptadora ASC y la caspasa-1 no mostraron diferencias significativas entre los grupos de estudio (figura 2A-D).

En la cuantificación de la expresión génica de las citocinas reguladoras IL- 10 y TGF-β, se encontró un aumento estadísticamente significativo del TGF-β en los grupos C y PT en comparación con el grupo P (p<0,001 y p<0,01, respectivamente), y de IL-10 en el grupo P en comparación con el grupo PT (p>0,05) (figura 2E-F).

### Perfil lipídico

**Figura 3:**
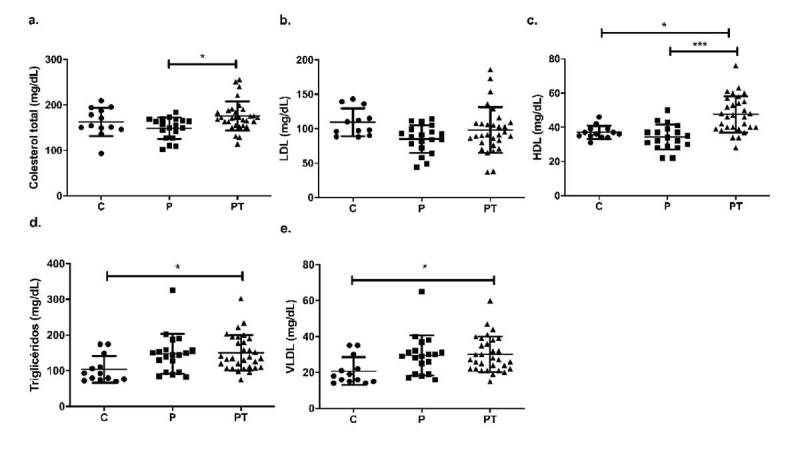
Perfil lipídico

**Figura 4:**
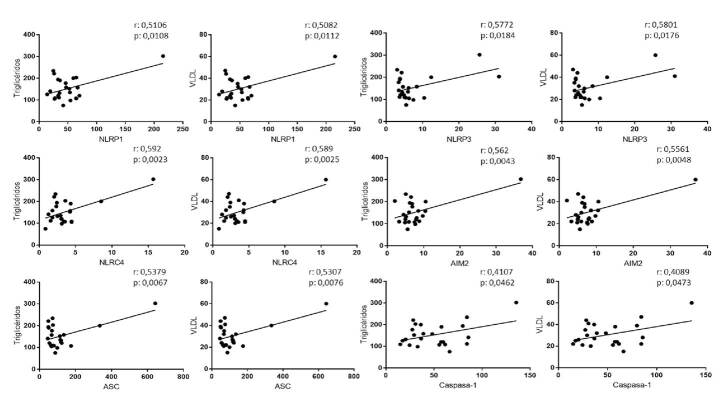
Correlación entre los valores del perfil lipídico y los componentes del inflamasoma en pacientes sin control de la replicación viral y con terapia, y correlación entre NLRP1, NLRP3, NLRC4, AIM2, ASC y caspasa-1 con triglicéridos y VLDL. Se utilizaron las pruebas de Pearson y Spearman, según la normalidad de los datos. El valor de p y r se indican en cada figura. Se consideraron estadísticamente significativos los valores de p<0,05.

Se determinaron los marcadores de riesgo cardiovascular estandarizados como colesterol total, HDL, LDL, VLDL y triglicéridos. Resulta interesante señalar que el grupo de individuos PT exhibía mayores niveles séricos de: colesterol total que el grupo P (mediana: 172 mg/dl *Vs*. 155 mg/dl; p<0,05); de triglicéridos que el grupo C (mediana: 135 mg/dl *Vs*. 92 mg/ dl; p<0,05); de HDL que los grupos P (mediana: 46,5 mg/dl *Vs*. 34 mg/dl; p<0,001) y C (mediana: 46,5 mg/dl *Vs*. 36 mg/dL; p<0,05), y de VLDL que el grupo C (mediana: 38 mg/dl *Vs*. 26 mg/dl; p<0,05) (figura 3). Al notar las diferencias estadísticamente significativas en la evaluación del perfil lipídico, especialmente de triglicéridos, VLDL y HDL, con los resultados notoriamente mayores en el grupo PT comparado con los otros dos grupos de estudio, se hicieron análisis de correlación con la expresión de los componentes de los inflamasomas, en especial con los niveles de triglicéridos y VLDL, pues al aumentar representan un mayor riesgo cardiovascular ^29^. Se evidenciaron correlaciones positivas entre los niveles de triglicéridos y las VLDL con los componentes de los inflamasomas (figura 4).

Los resultados de las mediciones de otros marcadores de riesgo cardiovascular, como la proteína C reactiva ultrasensible, el dímero D, la creatinina y otros marcadores inflamatorios como sCD14, sCD163 y la IL-6, no arrojaron diferencias estadísticamente significativas entre los grupos de comparación (figura suplementaria 1). También, se hicieron

correlaciones entre los niveles de creatinina en sangre y los componentes de los inflamasomas en el grupo de individuos PT, hallándose una correlación negativa entre la tasa de función renal y la expresión de dichas moléculas (NLRP3, NLRP4, ASC e IL-1β) (figura suplementaria 2).

## Discusión

En la literatura se ha descrito el papel que juegan las alteraciones metabólicas como factor de riesgo en el desarrollo de enfermedades cardiovasculares. Uno de los mecanismos involucrados en estas afecciones es la formación de ateromas, los cuales disminuyen progresivamente el calibre intraluminal de los vasos sanguíneos, favoreciendo su taponamiento e incrementando el riesgo de episodios coronarios agudos debido a la formación de trombos ^30^, muy asociada con la reacción inflamatoria, y el papel crucial de los inflamasomas en la fisiopatología de estas alteraciones ^14^^,^^31^^,^^32^.

Por ello, en el presente estudio, se exploró la relación entre algunos marcadores metabólicos y de riesgo cardiovascular y los componentes de los inflamasomas y moléculas involucradas en su activación en individuos con control de la replicación del HIV-1 y sin control de ella, con terapia antirretroviral o sin ella. Nuestros resultados evidenciaron que los individuos sin control de la replicación viral y con terapia antirretroviral exhibían un aumento en la expresión de los componentes de los inflamasomas, lo cual se correlacionó con los niveles de triglicéridos y VLDL, y sugiere un mayor riesgo de enfermedades cardiovasculares en estos pacientes. En estudios previos, también se ha demostrado aumento en la expresión de los componentes de los inflamasomas en pacientes que recibían terapia antirretroviral combinada ^33^ e, incluso, la activación del inflamasoma inducida por inhibidores de la transcriptasa inversa como el abacavir ^34^.

Los inflamasomas participan en la maduración de la IL-18 y la IL-1β ^20^^,^^35^, las cuales juegan un papel crucial en la generación de células espumosas derivadas de macrófagos o células endoteliales. Dichos efectos proinflamatorios pueden ser modulados por las HDL ^36^. Sin embargo, las células espumosas aumentan el reclutamiento de células inflamatorias alrededor de las placas ateromatosas en las paredes internas de las arterias, favoreciendo el desarrollo de enfermedades cardiovasculares ^37^.

Cabe resaltar que el grupo de pacientes sin control de la replicación viral y con terapia antirretroviral mostró un aumento significativo en la expresión de TLR2 y TLR4, lo que sugiere que, a pesar del adecuado control virológico inducido por la terapia antirretroviral combinada, en ellos se mantiene un ambiente proinflamatorio ^38^, posiblemente porque el tiempo de diagnóstico (y probablemente de infección) fue mayor en ellos, o por la influencia de los fármacos antirretrovirales ^39^^,^^40^.

Aunque en nuestro estudio no se encontraron diferencias significativas en la expresión de la proteína adaptadora ASC entre los grupos de estudio, en análisis previos de personas con infección por HIV-1, se ha reportado una mayor expresión de esta proteína, la cual participa en el desarrollo de la respuesta inflamatoria ^17^. Estos datos contrastantes podrían deberse al limitado tamaño muestral o a diferencias no exploradas aquí.

Por otra parte, una vez se logra controlar la replicación viral mediante la terapia antirretroviral combinada, las enfermedades cardiovasculares se convierten en la mayor causa de morbilidad y mortalidad en las personas con infección por HIV-1 ^41^, lo que no depende solamente de los mecanismos proinflamatorios ya descritos, sino también, de otros factores como el tabaquismo, las alteraciones endocrino-metabólicas, la hipertensión arterial sistémica y los diferentes esquemas de terapia antirretroviral combinada ^42^. De hecho, en los individuos PT se observó una correlación positiva entre los niveles de triglicéridos y de VLDL con componentes inflamatorios como la expresión de los inflamasomas NLRP1, NLRP3, NLRC4, AIM2 y otros asociados, como la proteína adaptadora ASC y la caspasa-1. Estos resultados podrían explicarse por la capacidad que tiene el HIV-1 de activar las cascadas moleculares de la inflamación, lo que genera alteraciones en el metabolismo de los lípidos y, en consecuencia, un aumento del riesgo cardiovascular ^12^^,^^43^.

Por otra parte, en el grupo sin control de la replicación viral y sin terapia se observó disminución de la expresión del TGF-β en comparación con los otros dos grupos, lo cual podría deberse a alteraciones asociadas con la replicación viral. Sin embargo, en otros estudios se han reportado niveles de TGF-β más altos en las personas con infección por HIV-1-sida antes de iniciar la terapia antirretroviral combinada, que disminuyen luego de 6 a 12 meses de tratamiento ^44^. Esta citocina tiene un importante papel antiaterosclerótico y su neutralización en modelos de ratón ha favorecido la pérdida de estabilidad de las placas ateromatosas, la cual incrementa el riesgo de enfermedades cardiovasculares ^45^.

Por otra parte, la expresión de la IL-10 fue significativamente menor en el grupo PT que en el P. En estudios previos, se ha demostrado que los niveles de esta citocina no guardan relación con la terapia antirretroviral combinada o el recuento de linfocitos T CD4+ ^46^ y, aunque se ha descrito su papel protector para evitar la formación de placas ateromatosas en modelos *in vitro*^47^, en estudios más recientes se ha asociado con un aumento del riesgo de enfermedades cardiovasculares ^48^^,^^49^.

Por último, aunque no se observaron diferencias significativas en parámetros clínicos asociados con el incremento del riesgo cardiovascular, tales como la proteína C reactiva ultrasensible, el dímero D, la creatinina y la glucemia en ayunas ^30^, en el perfil lipídico se encontraron niveles de colesterol total, VLDL y triglicéridos más altos en el grupo de pacientes sin control de la replicación viral y con terapia. Esto concuerda con estudios previos que muestran que, aunque las personas con infección por HIV-1- sida que reciben terapia antirretroviral combinada pueden tener un control virológico adecuado, persisten procesos activos de inflamación asociados a alteraciones en los niveles de los parámetros lipídicos que pueden incrementar el riesgo cardiovascular ^50^. Entre dichos mecanismos, se encuentran la activación inmunitaria persistente que favorece el desarrollo del síndrome metabólico, así como el tratamiento con algunos fármacos antirretrovirales, especialmente inhibidores de la proteasa, que afectan diferencialmente los niveles de colesterol y triglicéridos ^51^^-^^53^. Aunque sería interesante explorar esta asociación en nuestra población, en el estudio no fue posible estratificar el grupo de individuos PT en función de los diferentes esquemas disponibles de terapia antirretroviral combinada y el tiempo de tratamiento.

En conclusión, los pacientes sin control de la replicación viral y con terapia antirretroviral registraron un incremento en la expresión de los componentes de los inflamasomas, lo cual se correlaciona con los niveles de triglicéridos y de VLDL, y sugiere que factores como la activación inmunológica y la terapia antirretroviral están involucrados en el incremento del riesgo cardiovascular.

## Figures and Tables

**Figura S1:**
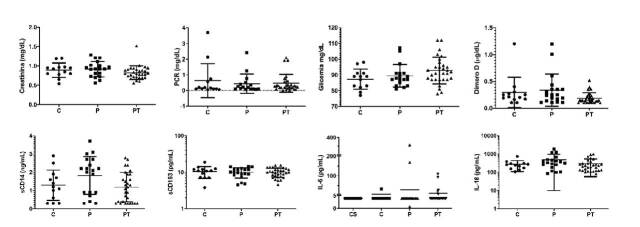
Cuantificación de otros marcadores metabólicos e inflamatorios: marcadores metabólicos (glucemia y creatinina) y de reacción inflamatoria (hs-PCR, dímero-D, sCD14, sCD163, IL-6 e IL-18). Las comparaciones se hicieron utilizando la prueba de Kruskal-Wallis y el test de Dunn post hoc. Todos los valores de p fueron superiores a 0,05.

**Figura S2:**
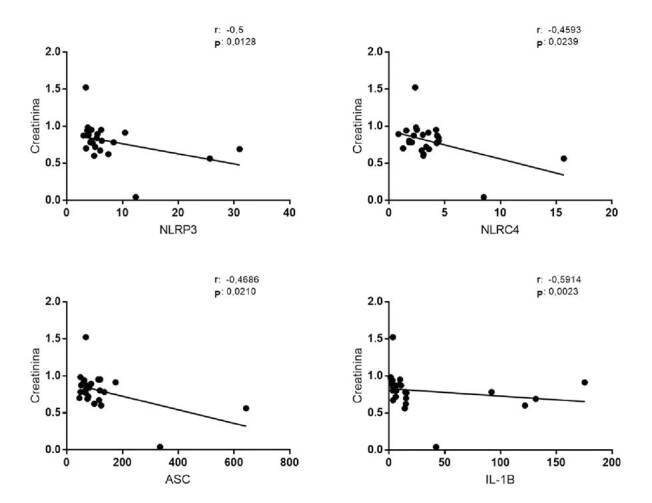
Correlación entre los niveles de creatinina y los componentes del inflamasoma pacientes sin control de la replicación viral y con terapia: correlación entre NLRP3, NLRC4, ASC e IL-1β y creatinina. Las correlaciones se evaluaron con las pruebas de Pearson y Spearman según la normalidad de los datos. Los valores de p y r se indican en cada figura; se consideró significativo un valor de p<0,05.

## Archivos suplementarios

**Cuadro S1:** Secuencia de los iniciadores empleados y las temperaturas de hibridación

Gen	Secuencia de iniciadores 5’-3’	Temperatura de hibridación (º C)
IL-1β	Fw: CTTTGCCGATCCGCCGC Rv: ATCACGCCCTGGTGCCTGG	60
IL18	Fw: ATGGCTGCTGAACCAGTAGAAG Rv: CAGCCATACCTCTAGGCTGGC	62
NLRP3	Fw: AGCACCAGCCAGAGTCTAAC Rv: CCCCAACCACAATCTCCGAAT	57
NLRP1	Fw: CTATACTTCCCGAGGCATCCTT Rv: GGTCTTGGAAGTCAGTGTGAGT	56
NLRC4	Fw: CTCTCATGGTGGAAGCCAGTCC Rv: ACAGAGACTTGACTATGTAATCC	56
AIM2	Fw: AAGCGCTGTTTGCCAGTTAT Rv: CACACGTGAGGCGCTATTTA	55
ASC	Fw: AACCCAAGCAAGATGCGGAAG Rv: TTAGGGCCTGGAGGAGCAAG	62
Caspasa-1	Fw: CAAGGGTGCTGAACAAGG Rv: GGGCATAGCTGGGTTGTC	60
TLR2	Fw: GGCCAGCAAATTACCTGTGTG Rv: CCAGGTAGGTCTTGGTGTTCA	63
TLR4	Fw: CTGCAATGGATCAAGGACCA Rv: TCCCACTCCAGGTAAGTGTT	61
β-actina	Fw: CTTTGCCGATCCGCCGC Rv: ATCACGCCCTGGTGCCTGG	60
TGF-β	Fw: CAGCAACAATTCCTGGCGATA Rv: AAGGCGAAAGCCCTCAATTT	60
IL-10	Fw: GCTGAGAACCAAGACCCAGAC Rv: GGAAGAAATCGATGACAGCG	60
